# Prognostic roles of mRNA expression of notch receptors in non-small cell lung cancer

**DOI:** 10.18632/oncotarget.14483

**Published:** 2017-01-04

**Authors:** Jianwen Xiong, Xiaoqiang Zhang, Xianglai Chen, Yiping Wei, De-guo Lu, Yun-wei Han, Jianjun Xu, Dongliang Yu

**Affiliations:** ^1^ Department of Cardiothoracic Surgery, The Second Affiliated Hospital of Nanchang University, Nanchang, Jiangxi Province 330006, P. R. China; ^2^ Department of Oncology, The Affiliated Hospital of Southwest Medical University, Luzhou, Sichuan 646000, China

**Keywords:** NSCLC, notch, hazard ratio, prognosis, KM plotter

## Abstract

Notch signalling is aberrantly activated in human non-small cell lung cancer (NSCLC). Nevertheless, the prognostic roles of mRNA expression of four Notch receptors in NSCLC patients remain elusive. In this report, we reported the prognostic roles of Notch receptors in a total of 1,926 NSCLC patients through “The Kaplan-Meier plotter” (KM plotter) database which is capable to assess the effect of 22,277 genes on survival of NSCLC patients. We found that mRNA high expression level of *Notch1* was associated with better overall survival (OS) for all NSCLC patients, hazard ratio (HR) 0.78 (0.69-0.89), *p*=0.00019, better OS in adenocarcinoma (Ade) patients, HR 0.59 (0.46-0.75), *p*=1.5e-05, as well as in squamous cell carcinoma (SCC) patients, HR 0.78 (0.62-0.99), *p*=0.044. mRNA high expression levels of *Notch2* and *Notch3* were associated with worsen OS for all NSCLC patients, as well as in Ade, but not in SCC patients. mRNA high expression level of *Notch4* was not found to be associated with to OS for all NSCLC patients. In addition, mRNA high expression levels of *Notch2, Notch3*, but *Notch4* are significantly associated with the NSCLC patients who have different smoking status. Our results indicate that mRNA expression of Notch receptors may have distinct prognostic values in NSCLC patients. These results will benefit for developing tools to accurately predict the prognosis of NSCLC patients.

## INTRODUCTION

Lung carcinoma is the leading cause of cancer-related death worldwide, and among this carcinoma, non-small cell lung carcinoma (NSCLC) comprises the majority of cases [1–2]. NSCLC includes: adenocarcinoma (Ade), squamous cell carcinoma (SCC), two major histological types. In spite of the progresses in early diagnosis, radical cure treatment, and molecular targeted therapies, the majority of NSCLC patients present with advanced stage disease and recur in about one third of individuals [3–4]. Therefore, it is still needed to further investigate the molecular mechanism of initiation, development and to identify potential prognostic markers and potential drug targets of NSCLC.

The Notch signaling pathway, an evolutionarily conserved and complex signaling system is known to regulate epithelial cell and stem cell homeostasis [5–6]. Notch signalling is aberrantly activated in several human tumors including breast, colon, cervical, pancreatic, head and neck, prostate cancer, renal carcinoma, Large-cell and Hodgkin lymphomas, as well as NSCLC [7–10]. Previous reports demonstrate that Notch signaling is activated and plays a critical role in NSCLC initiation, progression, and metastasis of NSCLC [10–13]. However, the prognostic values of mRNA expression of Notch receptors in NSCLC patients have not been determined. In this report, we determined the prognostic roles of mRNA expression of four Notch receptors in NSCLC patients.

The “Kaplan-Meier plotter” (KM plotter), established using data from Gene Expression Omnibus database was widely used in many studies [14–22]. A number of genes, as prognostic markers or potential drug targets have been reported by using KM plotter in ovarian cancer [23–24], breast cancer [25–33], gastric cancer [21, 34], as well as in NSCLC [35]. In this report, we used KM plotter database and determined the prognostic roles of mRNA expression of Notch receptors in NSCLC patients.

## RESULTS

It is well known that Notch signaling have four receptors, Notch1~4. All Notch receptors can be found in the database (www.kmplot.com).

**Figure 1:**
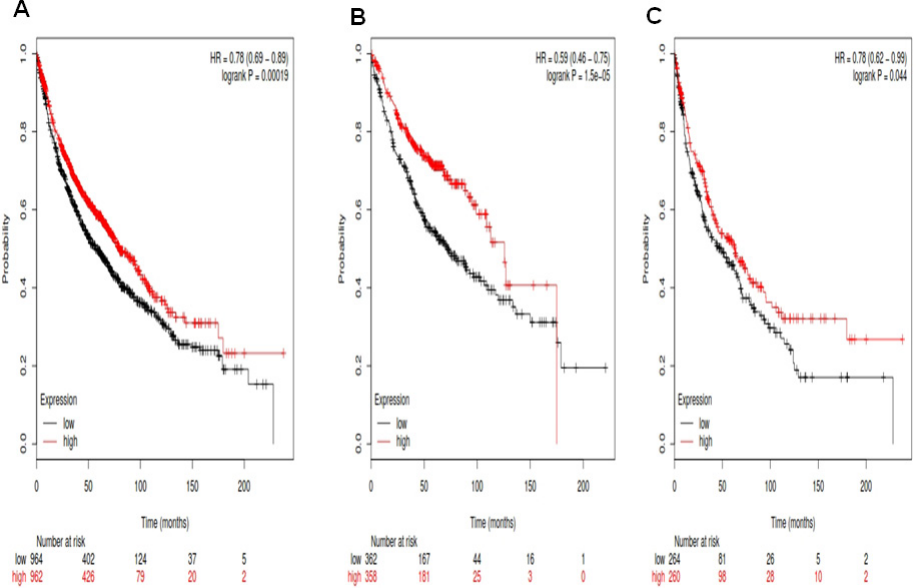
Determination of prognostic value of *Notch1* mRNA expression in the database The Affymetrix IDs is valid: 218902_at (*Notch1*). **A**. Survival curves are plotted for all NSCLC patients (n =1,926). **B**. Survival curves are plotted for adenocarcinoma (n =720). **C**. Survival curves are plotted for squamous cell carcinoma (n =524).

We used KM plotter and determined theprognostic value of *Notch1* in the database. The Affymetrix IDs is valid: 218902_at (*Notch1*). Survival curves are drafted for all NSCLC patients (n=1,926) (Figure 1A), for Ade patients (n =720) (Figure 1B), and for SCC patients (n =524) (Figure 1C). *Notch1* mRNA high expression was correlated to better overall survival (OS) for all NSCLC patients who were followed for 20 years, hazard ratio (HR) 0.78 (0.69-0.89), *p*=0.00019. *Notch1* mRNA high expression was also correlated to better OS in Ade patients, HR 0.59 (0.46-0.75), *p*=1.5e-05, as well as in SCC patients, HR 0.78 (0.62-0.99), *p*=0.044.

**Figure 2:**
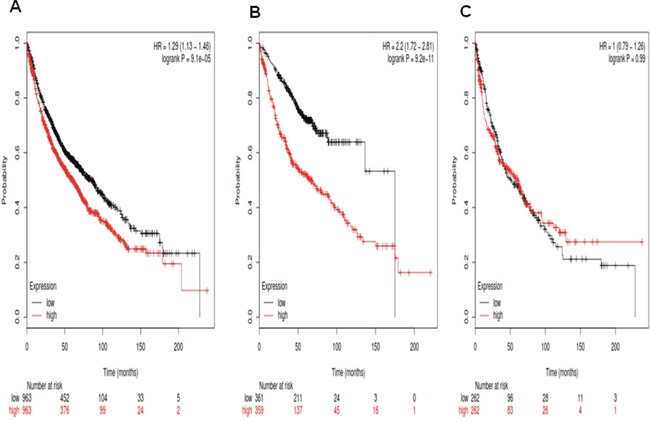
Determination of prognostic value of *Notch2* mRNA expression in the database The Affymetrix IDs is valid: 210756_s_at (*Notch2*). **A**. Survival curves are plotted for all NSCLC patients (n =1,926). **B**. Survival curves are plotted for adenocarcinoma (n =720). **C**. Survival curves are plotted for squamous cell carcinoma (n =524).

We next determined the prognostic value of *Notch2* in the database. The Affymetrix IDs is valid: 210756_s_at (*Notch2*). *Notch2* mRNA high expression was significantly correlated to worsen OS for all patients, HR 1.29 (1.13-1.46), *p*=9.1e-05 (Figure 2A). *Notch2* mRNA high expression was also correlated to worsen OS in Ade patients, HR 2.2 (1.72-2.81), *p*=9.2e-11 (Figure 2B), but not in SCC patients, HR 1 (0.79-1.26), *p*=0.99 (Figure 2C).

**Figure 3:**
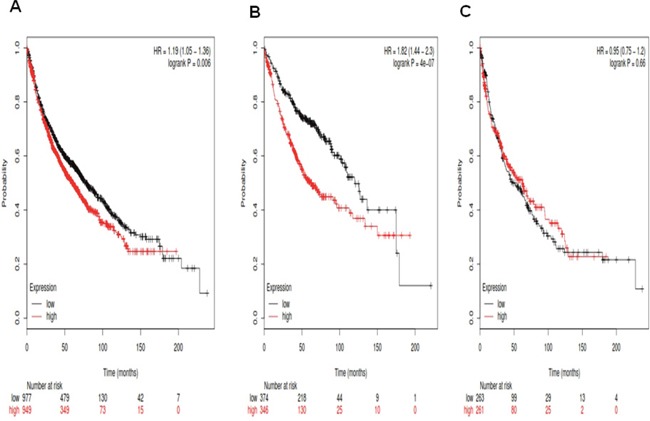
Determination of prognostic value of *Notch3* mRNA expression in the database The Affymetrix IDs is valid: 203237_at (*Notch3*). **A**. Survival curves are plotted for all NSCLC patients (n =1,926). **B**. Survival curves are plotted for adenocarcinoma (n =720). **C**. Survival curves are plotted for squamous cell carcinoma (n =524).

Figure 3 demonstrates the prognostic value of *Notch3* in the database. The Affymetrix IDs is valid: 203237_at (*Notch3*). *Notch3* mRNA high expression was significantly correlated to worsen OS for all patients, HR 1.19 (1.05-1.36), *p*=0.006 (Figure 3A) and Ade patients, HR 1.82 (1.44-2.3), *p*=4e-07 (Figure 3B), but not SCC patients, HR 0.95 (0.75-1.2), *p*=0.66 (Figure 3C).

**Figure 4:**
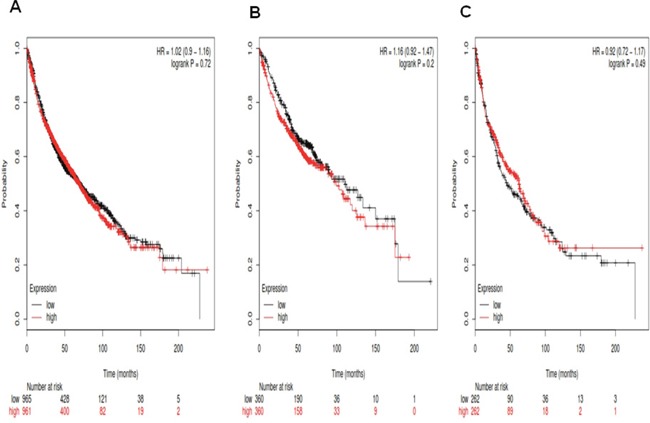
Determination of prognostic value of *Notch3* mRNA expression in the database The Affymetrix IDs is valid: 205247_at (*Notch4*). **A**. Survival curves are plotted for all NSCLC patients (n =1,926). **B**. Survival curves are plotted for adenocarcinoma (n =720). **C**. Survival curves are plotted for squamous cell carcinoma (n =524).

Figure 4 demonstrates the prognostic value of *Notch4* in the database. The Affymetrix IDs is valid: 205247_at (*Notch4*). *Notch4* mRNA high expression was not significantly correlated to OS for all NSCLC patients, HR1.02 (0.9-1.16), *p*=0.72 (Figure 4A), Ade patients, HR 1.16 (0.92-1.47), *p*=0.2 (Figure 4B), as well as patients with SCC, HR 0.92 (0.72-1.17), *p*=0.49 (Figure 4C).

For further assess the association of Notch receptors with other clinicopathological profiles, we determined the correlation with the patients' smoking status (Table 1), clinical different stages (Table 2) and different chemotherapeutic treatment (Table 3). As from Table 1, Notch2, Notch3, but Notch4 are significantly correlated with the smoking status. Notch 1 only significantly associated with smoked NSCLC patients. From Table 2, Notch1 and Notch2 are significantly correlated with clinical stage I & II of the patients. Notch 3 is only significantly associated with clinical stage I of NSCLC patients. From Table 3, only Notch3 is significantly correlated with the patients without chemotherapy.

**Table 1:** Correlation of Notch receptor mRNA expression with smoking status of NSCLC patients

Notch receptors	Smoking status	Cases	HR	95% CI	P value
Notch1	Never smoked	205	0.6	(0.34 - 1.06)	0.067
	Smoked	820	0.71	(0.58- 0.88)	0.0016
Notch2	Never smoked	205	2.97	(1.6 - 5.52)	0.00031
	Smoked	820	1.43	(1.16-1.76)	0.00091
Notch3	Never smoked	205	2.85	(1.57 - 5.17)	0.00032
	Smoked	820	1.41	(1.15 - 1.74)	0.001
Notch4	Never smoked	205	1.03	(0.59 - 1.8)	0.9
	Smoked	820	1.22	(0.99 - 1.49)	0.064

**Table 2:** Correlation of Notch receptor mRNA expression with clinical stages of NSCLC patients

Notch receptors	Clinical stages	Cases	HR	95% CI	P value
Notch 1	I	577	0.54	(0.41 - 0.71)	1e-05
	II	244	0.52	(0.35 - 0.75)	0.00042
	III	70	1.04	(0.6-1.79)	0.89
Notch 2	I	577	2.09	(1.58 - 2.77)	1.3e-07
	II	244	1.77	(1.22 - 2.56)	0.0022
	III	70	0.86	(0.5-1.49)	0.6
Notch 3	I	577	1.8	(1.37 - 2.36)	1.9e-05
	II	244	1.41	(0.98 - 2.04)	0.063
	III	70	0.93	(0.54-1.6)	0.8
Notch 4	I	577	0.93	(0.71 -1.21)	0.57
	II	244	0.91	(0.63 - 1.31)	0.61
	III	70	0.77	(0.45-1.32)	0.34

**Table 3:** Correlation of Notch receptor mRNA expression with chemotherapy of NSCLC patients

Notch receptors	Chemotherapy	Cases	HR	95% CI	P value
Notch1	No	310	1.08	(0.77 - 1.51)	0.66
	Yes	176	0.76	(0.5- 1.16)	0.21
Notch2	No	310	1.11	(0.8 - 1.56)	0.53
	Yes	176	0.75	(0.49-1.16)	0.19
Notch3	No	310	1.4	(1 - 1.97)	0.048
	Yes	176	0.76	(0.51 - 1.15)	0.19
Notch4	No	310	1.18	(0.85 - 1.66)	0.32
	Yes	176	0.8	(0.54 - 1.2)	0.29

## DISCUSSION

KM plotter was initially formed to access the prognostic value of an individual gene using online database in breast cancer patients [36–37]. A number of genes, such as ABCC2, BUBR1, CCND2, CCND3, CCNDE2, GREB1, MKI67, TK2, CDKN1A, TOP2A and TOP2B used this tool to validate or confirm the prognostic power in breast cancer [25–31, 38] and/or lung cancer [35]. In addition, several proteins were identified by KM plotter as potential drug targets, such as S100P [29], carbonic anhydrase IX (CAIX) [32], Notch1 [39] in breast cancer, aldehyde dehydrogenase 1 (ALDH1) [40] in NSCLC, Notch2 [24] in ovarian cancer.

Notch1, among four Notch receptors, is the best studied in NSCLC carcinogenesis [41–44]. Increased frequency of Notch1 activation can induce lung adenoma formation and enable progression to Ade and metastases [45]. Notch1 activation can also enhance epithelial-mesenchymal transition (EMT) in lung cancer cells [46–47]. Cisplatin was also observed to activate Notch1 first, then further induce the enrichment of CD133 positive cells in lung Ade [48]. Notch1 function was found to be required for tumor initiation through suppression of p53-mediated apoptosis in NSCLC [49]. But recently, Wael et al observed that Notch1 expression has a tumor inhibitory effect on Ade cells, but not SCC cells in NSCLC [50]. A meta-analysis showed that Notch1 was correlated with lymph node metastasis, TNM stages and significantly poor OS in NSCLC patients [51]. *Notch1* mRNA was expressed on both mesenchymal and epithelial structures of the embryonic lung, indicating that Notch1 has a role in the vascular morphogenesis and lung development [52]. In our report, mRNA high expression of *Notch1* was correlated to better OS for all NSCLC patients, also better OS in Ade and SCC patients.

Notch2 is a tumor suppressor in lung carcinogenesis [53]. But, the prognostic significance of either Notch2 protein or mRNA in NSCLC patients is not known. In our report, mRNA high expression of *Notch2* was significantly correlated to worsen OS for all NSCLC patients, HR 1.29 (1.13-1.46), *p*=9.1e-05. *Notch2* mRNA high expression was also correlated to worsen OS in Ade patients, HR 2.2 (1.72-2.81), *p*=9.2e-11, but not in SCC patients, HR 1 (0.79-1.26), *p*=0.99.

Notch3 potentially contributes to the multistep evolution of lung cancer in airway epithelium by using a transgenic mouse model [54]. High Notch3 expression may contribute the resistance to chemotherapy in lung cancer patients [55]. Notch3 was found to upregulate ZEB-1, which leads to TGF-β-induced the EMT and bone metastasis in NSCLC [56]. A report showed that Notch3 was an independent prognostic factor for patients with NSCLC [57]. In consistent with previous report, our results showed that mRNA high expression of *Notch3* was found to be significantly correlated to worsen OS for all NSCLC patients, HR 1.19 (1.05-1.36), *p*=0.006 and Ade patients, HR 1.82 (1.44-2.3), *p*=4e-07, but not patients with SCC, HR 0.95 (0.75-1.2), *p*=0.66.

Notch4 is an oncogene in mammary tumor [58–59]. Notch4 was able to expended the tumorigenicity *in* mammary epithelial cells [60–61]. Although Notch4 was detected in both tumor and stromal compartments of NSCLC [62], the significance of Notch4 in NSCLC needs to be further determined. In this report, mRNA high expression of *Notch4* was not significantly correlated to OS for all NSCLC patients, Ade patients, as well as SCC patients.

Nicotine can deregulate essential biological activities such as the cell apoptosis, proliferation, migration, invasion, inflammation, cell-mediated immunity and angiogenesis [63]. Nicotine might be promoting NSCLC growth and metastasis by inducing the secretion of stem cell factor [64]. There is no report regarding whether there is a direct correlation between nicotine and Notch activation in NSCLC. However, Hirata et al [65] reported that nicotine can increase ALDH-positive cells through a PKC-Notch pathway in MCF-7 cells. In addition, nicotine may impact specific Notch members in endothelial cells [66] or dendritic cells [67], thus indirectly impact NSCLC. Nicotine was also reported recently to enhance the expression of synaptic and Notch1 proteins in stressed animals [68]. In this report, we observed that high mRNA expression of Notch2, Notch3, but Notch4 are correlated with smoking status of NSCLC patients. High mRNA expression of Notch 1 is only significantly associated with smoked NSCLC patients.

Previous results suggest that Notch signaling, especially Notch receptors may be essential drug target for NSCLC patients. γ-secretase inhibitor, DAPT was demonstrated to inhibit Notch activation and cell growth in NSCLC cells [69]. However, γ-secretase inhibitors are not able to distinguish individual Notch receptors and may cause intestinal toxicity [70] by inhibiting other signaling pathways [71]. Recently, phage display technology may produce highly specialized antibodies which can recognize each Notch receptor paralogue in human patients and rodent models [72]. Based on our study that mRNA high expression of *Notch2* and *Notch3* was correlated to worsen OS for all NSCLC patients, thus Notch2 and Notch3 might be potential drug targets for NSCLC patients.

In summary, we demonstrated that *Notch1* mRNA high expression was correlated to better OS for all NSCLC patients. *Notch2* and *Notch3* mRNA high expression were correlated to worsen OS for all NSCLC patients. *Notch4* mRNA high expression were not correlated to OS for all NSCLC patients. These results will be useful for better understand the complexity and heterogeneity in the molecular biology of NSCLC and to develop tools to more accurately predict their prognosis. Based on our study, Notch2 and Notch3 might be potential drug targets for NSCLC patients.

## MATERIALS AND METHODS

An online database [36] was used to determine the relevance and significance of Notch receptors' mRNA expression to overall survival (OS). NSCLC patients in the KM plotter database were come from the Gene Expression Omnibus (GEO, http://www.ncbi.nlm.nih.gov/geo/), Cancer Biomedical Informatics Grid (caBIG, https://biospecimens.cancer.gov/relatedinitiatives/overview/caBig.asp), and The Cancer Genome Atlas (TCGA, http://cancergenome.nih.gov) lung cancer datasets [73]. The database was established using gene expression data and survival information of 1,928 NSCLC patients downloaded from above three datasets. The NSCLC patients were followed up 20 years. By using KM plotter, four Notch receptors (*Notch1, Notch2, Notch3* and *Notch4)* were entered into the database (http://kmplot.com/analysis/index.php?p=service&cancer=breast) to obtain Kaplan-Meier survival plots in which the number-at-risk is indicated below the main plot. The certain gene mRNA expression above or below the median separates the cases into high expression and low expression. Hazard ratio (and 95% confidence intervals) and log rank P were calculated and displayed on the webpage.
